# Disclosing Topographical and Chemical Patterns in Confined Films of High-Molecular-Weight Block Copolymers under Controlled Solvothermal Annealing

**DOI:** 10.3390/polym16131943

**Published:** 2024-07-08

**Authors:** Xiao Cheng, Jenny Tempeler, Serhiy Danylyuk, Alexander Böker, Larisa Tsarkova

**Affiliations:** 1 Fraunhofer Institute for Applied Polymer Research (IAP), Geiselbergstr. 69, 14476 Potsdam-Golm, Germany; xcheng_21@seu.edu.cn (X.C.); alexander.boeker@iap.fraunhofer.de (A.B.); 2 School of Civil Engineering, Southeast University, Dongnandaxue Road 2, Jiangning District, Nanjing 211189, China; 3 Fraunhofer Institute for Laser Technology (ILT), Steinbachstr. 15, 52074 Aachen, Germany; jenny.tempeler@trumpf.com (J.T.); serhiy.danylyuk@ilt.fraunhofer.de (S.D.); 4 German Textile Research Center North-West (DTNW), Adlerstr. 1, 47798 Krefeld, Germany

**Keywords:** block copolymer films, solvothermal annealing, high molecular weight, confined self-assembly

## Abstract

The microphase separation of high-molecular-weight block copolymers into nanostructured films is strongly dependent on the surface fields. Both, the chain mobility and the effective interaction parameters can lead to deviations from the bulk morphologies in the structures adjacent to the substrate. Resolving frustrated morphologies with domain period L_0_ above 100 nm is an experimental challenge. Here, solvothermal annealing was used to assess the contribution of elevated temperatures of the vapor T_v_ and of the substrate T_s_ on the evolution of the microphase-separated structures in thin films symmetric of polystyrene-b-poly(2vinylpyridine) block copolymer (PS-PVP) with L_0_ about 120 nm. Pronounced topographic mesh-like and stripe patterns develop on a time scale of min and are attributed to the perforated lamella (PL) and up-standing lamella phases. By setting T_v_/T_s_ combinations it is possible to tune the sizes of the resulting PL patterns by almost 10%. Resolving chemical periodicity using selective metallization of the structures revealed multiplication of the topographic stripes, i.e., complex segregation of the component within the topographic pattern, presumably as a result of morphological phase transition from initial non-equilibrium spherical morphology. Reported results reveal approaches to tune the topographical and chemical periodicity of microphase separation of high-molecular-weight block copolymers under strong confinement, which is essential for exploiting these structures as functional templates.

## 1. Introduction

Among the variety of nanofabrication bottom-up methods, directed assembly of microphase-separated structures of block copolymers is recognized for its effectiveness, low cost and flexibility in producing large-area films with well-defined and well-ordered nanostructures [1,2,3,4]. Fabrication of patterns with periodic features above 100 nm has drawn growing attention, driven by potential applications, e.g., in photonics, reflective coatings, and interconnect patterning [5,6,7,8,9,10,11,12,13,14]. Such large feature sizes are achieved by employing high-molecular-weight block copolymers, in the range around or above 500 kg/mol. Accordingly, new synthetic approaches to achieve ultralong linear chains [15,16,17] or more complex architectures [14,18,19,20,21] have been reported. The processing of such long chains into functional materials or films raises a number of fundamental and technological issues that have been analyzed in detail earlier [1,22]. High-molecular-weight block copolymers exhibit high viscosity, even in a swollen state, as a result of chain entanglements, leading to long processing times and uncompleted microphase separation [15,22]. Chain mobility is also strongly dampened by multiple contacts of long chains with the substrate, leading to a strong pinning to the substrate and therefore promoting the formation of “asymmetric” structures at the substrate and at the free surface of the film [23,24]. The resolution of the frustrated morphologies, caused by the strong surface fields, is an experimental challenge.

To overcome the processing difficulties, researchers employ various strategies, such as different solvent vapor annealing methods [25,26,27,28,29,30,31,32,33,34,35], including swelling to high ratios by using appropriate solvents/mixtures of solvents [36,37], or overpressure [38], adding non-volatile solvent during film casting [39], using homopolymers as smart solvents and compatibilizers [26,40,41], employing swelling network supports [42] or non-solvent immersion treatments [43]. Despite these efforts, it is clear that achieving long-range orientational order and defectless structures is not feasible for high-molecular-weight block copolymers [44]. Therefore, the potential is seen in applications that rely on nanostructures with dimensions in the optical range, such as hierarchical optical metasurfaces [45], mesoporous materials [46,47], including hybrid inorganic materials based on selective infiltration of block copolymer templates [48,49]. Taking into account the high sensitivity of the microphase separation to the small variations in the film thickness, solvent selectivity, and interactions with the substrate, modern machine learning design approaches can be applied to relate the processing conditions to the defectivity of the resulting patterns [50].

Solvent vapor annealing has the advantage of reducing interactions of polymers with the substrate, thus eliminating the pinning effect and significantly reducing annealing time [35]. Solvent molecules affect polymer-polymer interaction parameters, which in the case of block copolymer solutions is referred to as χ_eff_ [51]. Theoretically ϕ_eff_ in block copolymer solutions has been elaborated by Fredrickson and Leibler [52] and Olvera de la Cruz [53] and extended later by Lodge [51]. Furthermore, the effective changes to the polymer volume fraction as a result of selective swelling [54] can induce morphological changes [55]. A comprehensive analysis of solvent vapor processing of high-molecular-weight block copolymers was recently given by Mokarian-Tabari and co-workers [37]. The authors reported enhanced ordering on a timescale of 10 min of perforated lamella structures with a domain period of about 190 nm by fast swelling of polystyrene-*b*-poly(2vinylpyridine) films to very high levels of solvent concentration. Although this work demonstrated the importance of processing parameters, such as the swelling rate, hold times, and the polymer concentration in the swollen film (φ_p_), the influence of the absolute temperatures of the vapor and of the substrate in this processing remains predominantly unexplored.

Solvothermal annealing is envisaged to offer additional advantages in processing high-molecular-weight polymers, such as enhanced ordering dynamics, improved long-range order and tunable morphologies by careful selection of solvents and annealing conditions [28,54]. However, introducing additional variables in the process makes the understanding of the self-assembly mechanisms under solvothermal annealing and identification of the microphase-separated patterns increasingly complicated, because both temperature and solvent affect the segmental mobility, molecular interaction parameters, solubility and solvent quality, as well as surface/interfacial tension in films. Since the intrinsic domain spacings L_0_ of high-molecular-weight block copolymers are larger than 100 nm, all films with a swollen thickness below L_0_ belong to a category of highly frustrated microphase-separated structures, which deviate from bulk morphologies. Further challenges are associated with the estimates of the interaction parameter χ under swollen conditions. Using a classical equation for bulk χ_eff_ = χφ_p_^b^ (where χ_eff_ and χ are Flory–Huggins interaction parameters in swollen and dry films, respectively, b is an exponent factor, which varies between 1 and 2) [51] is not straightforward as it does not take into account polymer-substrate interactions. Furthermore, even a slight selectivity of the solvent to the substrate or to the block copolymer components can cause significant changes in the symmetry or dimensions of the resulting pattern.

Although hot/warm solvent vapor annealing has been used in previous studies [54,56,57], the competitive influence of the annealing temperatures on the variation in χ and other enthalpic parameters in the system, as well as on the solvent uptake and chain dynamics has not systematically evaluated, both in terms of pattern tunability and in terms of enhancing segmental dynamics.

Here we resolve mesoscale topographic and chemical patterns in sub 100 nm films of high-molecular-weight polystyrene-b-poly(2vinylpyridine) (PS-PVP) diblock copolymer subjected to temperature-controlled solvent vapor annealing in a specially designed annealing cell [28]. We first analyze the resulting microphase-separated mesh-like and stripe patterns as a function of the temperatures of the vapors (T_v_) of the chloroform (a slightly selective solvent, Figure S1, Supplemental Information) and the polymer film (substrate, T_s_). The dynamics of the structure evolution upon varying the annealing time from minutes to hours is also discussed. Finally, we identify the match between the topographic and chemical (PS and PVP domains) patterns, which was explored using the floating technique, reactive ion etching (RIE) for pattern transfer, as well as by selective metallization of the structures.

## 2. Materials and Methods

### 2.1. Materials

Polystyrene-*b*-poly(2-vinyl pyridine) (denoted here as PS-PVP) diblock copolymer with a total molecular weight of M_n_ = 390 kg/mol and a volume fraction of PS block φ_ps_ = 0.48, as well as the homopolymer poly(2-vinyl pyridine) (PVP) (M_n_ = 105 kg/mol) were synthesized by anionic polymerization [58]. A degree of polymerization of PS-PVP of ~3609 and an interaction parameter χ_PS-PVP_ of ~ 0.178 at room temperature, resulting in a high segregation strength of χN ≈ 640. Domain spacing L_0_ of ~117 nm was determined by small angle X-ray scattering (SAXS) at the European Synchrotron Radiation Facility, Grenoble, France, by measuring solvent-cast µm-thick PS-PVP film (Figure S3, Supplemental Information). Polystyrene (PS) (M_n_ = 180 kg/mol) was purchased from PSS (Polymer Standards Service, Amherst, MA 01002, USA). Chloroform and toluene were purchased from Sigma-Aldrich (Sigma-Aldrich Chemie GmbH, Schnelldorf, Germany) and used without further purification.

### 2.2. Film Preparation

P-type Si wafers (Crys Tec GmbH, Berlin, Germany) with ~2 nm thick SiO_x_ layer were cut in pieces and stored in toluene. Before usage, silicon substrates were additionally cleaned by a CO_2_ snow-jet gun and then treated with air plasma at 60 W for 1 min. Films with a targeted thickness of below 50 nm have been prepared by spin-coating polymers from a toluene solution with a concentration of 1 wt%. Toluene is a selective solvent for the PS block. After spin-coating, the films were dried at room temperature in a vacuum oven for 12 h to remove the residual solvent.

### 2.3. Floating of the Films

First, a prepared film on the Si wafer was repeatedly touched on the surface of aqueous NaOH solution (1 mM) with the sample edge at ~45° until the block copolymer film was detached from the substrates and floated on the surface of the solution. To image the back side of the film, it was then picked up with a clean silicon wafer upside down, resulting in a strong adhesion of the picked-up film to the substrate [59]. The films were then rinsed in deionized water and placed in a vacuum oven for 12 h at ambient temperature to remove the residual moisture.

### 2.4. Annealing Experiments

**Figure 1:**
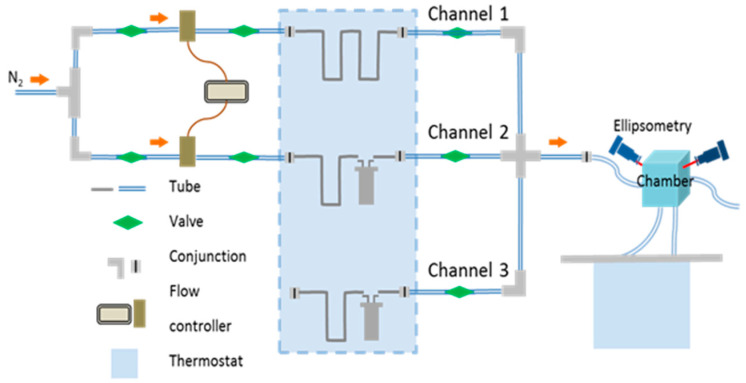
Sketch of the annealing set up. Reprinted from Ref [28] with permission.

Solvent annealing was performed in a custom-made setup (Figure 1). Nitrogen, as a carrier gas, passes through two flow controllers (MKS 647C, MKS Instruments Deutschland GmbH, München, Germany) connected to the channel with a flow of pure nitrogen (Channel 1) and to the channel that delivers solvent vapor (Channel 2) into the chamber. Flow controllers define the total flow through a channel (a maximum value of 100 sccm, unless otherwise specified), as well as the partial vapor pressure in the chamber p/p_0_. The latter is adjusted by mixing the flows through Channel 1 and Channel 2. The temperature of the vapor T_v_ was maintained by a bath thermostat with immersed vials with the solvent. The temperature of the substrate T_s_ was adjusted by cycling water through the tubing at the bottom of the chamber. Further details can be found in Ref [28].

### 2.5. Characterization of the Films

Swelling behavior of polymer films was monitored by in situ spectroscopic ellipsometry (Omt Imaging, mm30 series, Ulm, Germany). Optical data were collected within a spectral range of 450–800 nm at an incidence angle of 70° using VisuEl 3.8 software (Omt-optische messtechnik GmbH, Ulm, Germany). Film thicknesses were evaluated using the Cauchy model and Scout Software 4.7 (Omt-optische messtechnik GmbH, Ulm, Germany). The polymer fraction in a swollen film is presented as φ_p_, calculated by h_d_/h_sw_ where the h_d_ is the start film thickness and h_sw_ is the swollen film thickness. The degree of swelling D = 1/φ_p_.

Scanning force microscopy (SFM) was conducted using Icon Dimension (Bruker Corporation, Billerica, MA 01821, USA) in TappingMode using tips from OTESPA with spring constant k = 42 N/m. The images were analyzed using Nanoscope Analysis 1.50 software (Bruker Corporation, Billerica, MA 01821, USA).

### 2.6. Metallization of the Microphase Separated Structures

Pt^2+^ ions were loaded by immersion of the film into 1 wt% Na_2_PtCl_4_ solution in 5 mM HCl. PVP blocks become protonated in an acidic environment so that the Pt^2−^ anions build complexes with the cationic groups of the PVP blocks. The following treatment with oxygen plasma (under 0.2 mbar at 80 W for 90 s) removes the polymer, leaving on the substrate the metalized pattern, which can be assigned to the PVP block.

### 2.7. Pattern Transfer

Selected structures have been subjected to reactive ion etching (RIE) to demonstrate the applicability of the self-assembled block copolymer topographic structures for pattern transfer into the silicon substrate. The samples were etched in a CHF_3_/O_2_/SF_6_ plasma at a chamber pressure of 0.033 mbar and an RF power of 35 W with an average PVP etch rate of 28 nm/min and PS etch rate of 12 nm/min.

## 3. Results and Discussions

### 3.1. Swelling under Controlled Solvothermal Conditions

Swelling experiments under controlled flow and temperature conditions were carried out in a custom-built chamber using in situ ellipsometry measurements of the swollen film thickness, as briefly described in the Materials and Methods Section and more fully in the Supplemental Information. It is suggested, that maintaining the swollen block copolymer film at a slightly elevated temperature may improve the annealing dynamics through a combination of thermally enhanced segmental mobility and of solvent plasticizing effect. We note that a qualitative assessment of this hypothesis is not feasible due to the uncertainty in the interaction parameters, as mentioned in the introduction. In particular, the reduced pinning of the segments to the substrate due to the weakening of the surface interactions may be compromised by a reduced solvent uptake, when the temperature of the substrate is increased (Figure S2b). Furthermore, the combination of strong surface fields at the substrate and the solvent-induced shift in the interaction parameters can lead to the deviation of bulk morphologies. Also, solubility parameters are not easy to estimate because of the often-observed thickness-dependent swelling [60,61]. Another challenging experimental factor is the potential condensation of the solvent on the film surface as a result of the increased temperature of the vapor, i.e., of the solvent concentration in the vapors. To explore the possibilities of our annealing setup to run solvothermal annealing in a reproducible and controlled manner, we analyzed the response of the solvent uptake by PS-PVP films to the changes in the temperature of the vapors (T_v_) and of the polymer film (substrate, T_s_) (Figure S2, Supplemental information).

The choice of the film thickness in the swollen state below the characteristic lamella spacing of PS-PVP in L_0_ of 117 nm (Figure S3, Supplemental Information), resulting in frustrated morphologies, allowed a closer insight into the effect of the annealing parameters on the strength of the surface fields. It was shown that the development of specific defects or phase transitions as annihilation pathways toward equilibrium can be used to discuss the surface fields in the system [62]. We note, that in this particular system, as the swollen film thickness approaches L_0_ (by using thicker films or high degrees of swelling, as a result of solvent condensation) the formation of in-plane oriented lamella, i.e., featureless patterns, occurs (Figure S4, Supplemental Information) as a result of highly preferred interactions of the polar PVP block with the substrate.

### 3.2. Effect of Solvothermal Conditions and Annealing Time on the Microphase Separation Behavior of PS-PVP in Thin Films

**Figure 2:**
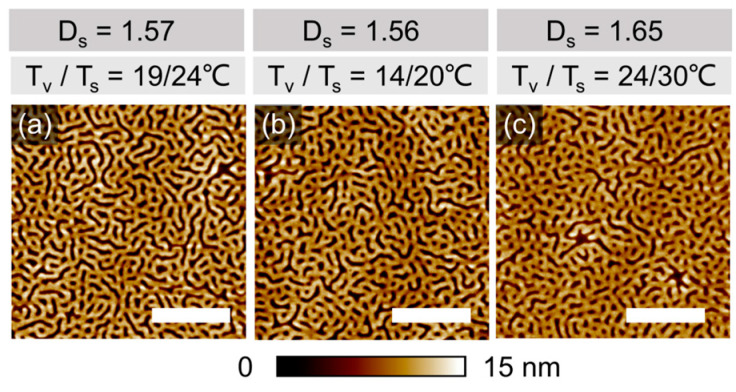
SFM topography images of PS-PVP films with *h_dry_*= 42 ± 2 nm annealed: (**a**–**c**) under indicated T_v_/T_s_ and 100% p/p_0_ of chloroform vapor for 10 min. D_s_ = h_sw_/*h_dry_* represents a degree of swelling. The scale bar in each image is 1 μm.

Shown in Figure 2a–c are topographic SFM images of PS-PVP films with an initially dry thickness of ~ 42 nm, well below the intrinsic domain spacing *L*_0_ = 117 nm, which were annealed for 10 min at different temperature sets T_v_/T_s_ with an intended ΔT = T_s_ − T_v_ difference of 5–6 °C. The desired precision in the targeted degree of swelling D_s_ was not feasible to achieve in different comparative experiments, since multiple dynamic and thermodynamic parameters affect the solvent uptake. However, we believe that the slight deviations in the annealing conditions can be tolerated for interpreting the developed structures.

**Figure 3:**
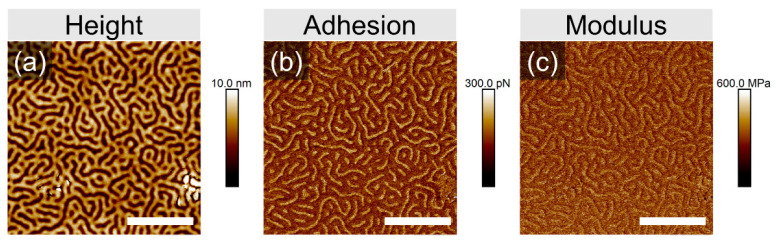
SFM height (**a**), adhesion (**b**) and modulus (**c**) images taken by PeakForce Tapping of PS-PVP film with a dry thickness of ~40 nm annealed in 100% *p*/*p*_0_ chloroform vapors at T_v_/T_s_ 25/26 °C. The white scale bar in each image corresponds to 1 μm.

The evolved structures are represented in each case by a disordered striped pattern, which can be assigned to up-standing lamella, since even in the swollen state the film thickness is still below L_0_. The differences in the degree of ordering, achieved within 10 min of annealing, or in the shape of specific defects are too marginal to be discussed. However, we elaborate on the origin of the pronounced topography of the pattern, which is likely due to the high selectivity of chloroform to the PVP block. The lower channels with a depth of 8 ± 1 nm can be assigned to de-swollen PVP domains, which in a swollen state accumulate about 15% more solvent than PS domains (Figure S1, Supplemental Information). This assumption is supported by the measurement in PeakForce Tapping mode, which provides space-resolved information about the adhesion force between the tip and the material, as well as the local modulus. Therefore, the brighter-color area implies a higher adhesion force to the tip (Figure 3b) and a higher modulus of the material (Figure 3c). The surface of the employed silicon nitride tip has a polar nature and therefore the tip adheres stronger to PVP domains than to PS domains. Also, in reported earlier phase images of PS-PVP block copolymers, the PVP domains appear brighter as a result of their slightly higher T_g_ as compared to PS [38]. Otherwise, we note that resolving the phase contrast in phase imaging often results in scanning artifacts (Figure S5, Supplemental Information), and resolving the chemical patterns is a separate challenge, which will be addressed later.

**Figure 4:**
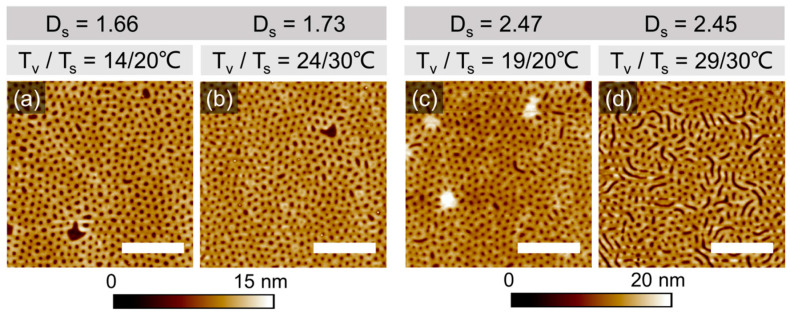
SFM topography images of PS-PVP films with *h_dry_*= 42 ± 2 nm annealed under T_v_/T_s_ of (**a**) 14/20 °C, (**b**)24/30 °C, (**c**) 19/20 °C, and (**d**) 29/30 °C with 100% *p*/*p*_0_ of chloroform vapor for 200 min. The corresponding D_s_ are located at the top of each image. *h_sw_* are ~70 nm and ~102 nm for *D_s_* ~1.7 and ~2.5, respectively. The white scale bar in each image corresponds to 1 μm. Evaluated domain spacings are shown in Table 1 (Figures S4 and S5, Supplemental Information).

Since 10 min-long annealing did not indicate the effect of the annealing conditions on the microphase separation of PS-PVP domains, below we consider the morphological evolution on a longer annealing time scale. SFM images in Figure 4 and Figure S6 (Supplemental Information) show representative surface structures in films of PS-PVP with *h_dry_* ~42 ± 2 nm, which have been processed under indicated solvothermal conditions for 200 min. The swelling conditions were chosen so that a comparable degree of swelling could be maintained under different temperature sets T_v_/T_s_.

The microphase-separated structure, which was developed in the swollen state, appears in the quenched state as a dot-like topographic pattern and can be attributed to the perforated lamella (PL) phase. The formation of this morphology is enabled both by the film thickness below L_0_ and by the selectivity of the solvent, which effectively shifts the initially symmetric volume composition of the block copolymer towards PVP being a majority block. PL is represented by two layers of swollen PVP block, which face both interfaces of the film and perforate the middle PS block. During rapid evaporation of the solvent, the film undergoes one-dimensional compression and the PVP perforations appear as holes (5–6 nm deep).

In all cases, the structures have a poor long-range order, confirming the well-established difficulties in processing high-molecular-weight block copolymers [22,27].

The structure in Figure 4d can be characterized as a coexistence of the PL and stripes of the vertically oriented lamella phase. This can be explained as an adjustment of the PL phase to a slight excess of the film thickness [63]. Further characteristic defects of the patterns in Figure 4 can be explained along the lines of the thickness-dependent morphological behavior, which will be reported in detail elsewhere (Figure S6, Supplemental Information).

The formation of the PL phase in ultrathin solvent-annealed confined films of high-molecular-weight PS-PVP is in agreement with an earlier study by Selkirk et al. [37], despite the fact that they employed a mixture of solvents, aiming at non-selective solvent conditions.

Quantitative evaluation of the dimensions of the patterns in Figure 4 was performed using PSD analysis (Figure S7, Supplemental Information). The depth of the patterns has been averaged over at least 10 cross-sectional profiles of the SFM scans. The center-to-center distances (CCD) of the periodic structures are summarized in Table 1. We believe that the scatter in the measured values is due to the intrinsically low mobility of high-molecular-weight block copolymers, and even more due to the confined geometry when the surface field dominates the microphase separation over the other thermodynamic parameters in the system (such as volume fraction, χ_eff_ interaction parameter, temperature). Despite significant deviations in the evaluated dimensions, it can be concluded that the size of the resulting patterns depends on the particular T_v_/T_s_ combination. The effect is less pronounced at higher degrees of swelling.

Because of prolonged annealing, we believe that the developed structures are in a (quasi)equilibrium state, i.e., the reasons behind the tunable structure dimensions are rather thermodynamic, than kinetic ones. Generally, the smaller the characteristic period of the domains, the larger the interface between the block copolymer components PS and PVP. The formation of a more extended PS/PVP interface at D_s_~1.7 at a higher temperature set of 24/30 °C (Table 1) is presumably due to effectively smaller χ interaction parameters under these conditions. Additional arguments in terms of the decreased surface/interfacial tensions with increasing temperature can also be considered. Likely, in the particular narrow parameter window that we have tested the enhanced chain mobility due to the elevated temperature of the substrate is not sufficient to overcome the pinning of the chains to the substrate. Rather, the microphase-separated structures undergo unit cell distortion because the surface fields still overcome the energy required to distort the structure. Furthermore, the selectivity of solvent can be affected by the specific temperature set, leading to changes in the preferential swelling of the blocks, and hence to effective shifting of the volume fraction.

The present paper suggests that by choosing a particular T_v_/T_s_ combination, it is possible to tune the sizes of the resulting PL patterns by almost 10%. In the following, we attempt to characterize the microphase-separated structures in confined PS-PVP films in more detail. In particular, we focus on establishing the correlation between the pronounced topographic pattern of the microstructures and the distribution of the blocks within the topographic features, taking advantage of the differences in the chemistry of the blocks.

### 3.3. Characterization of the Patterns

**Figure 5:**
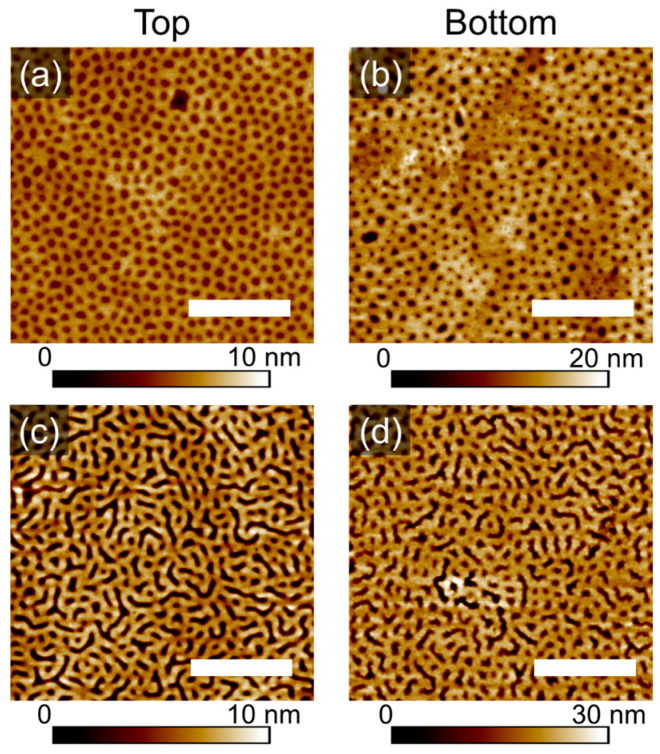

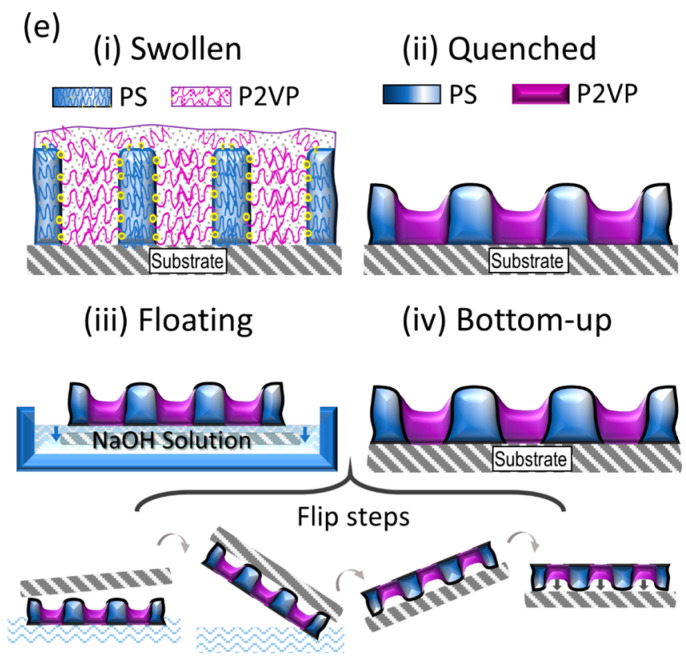
SFM topography images of the free surface (Top, **a**,**c**) of PS-PVP films after annealing and of the backward side of the same sample after floating and deposition on a new substrate (Bottom, **b**,**d**). The scale bar is 1 μm. (**e**) Schematic of the swollen and quenched film resulting in topographic pattern (i,ii), of the film floating (iii) and re-deposition on a new substrate (iv). The arrows in (iii) indicate the detachment of the film from the Si substrate to the surface of the NaOH solution, followed by the pick-up of the film bottom-up with a fresh substrate and the adhesion of the film.

Presented in Figure 5a–d are topography images of the dot-like and striped patterns in thin films of PS-PVP, which have been produced by scanning the free (top) surface of the films, as well as the surface next to the substrate. This was possible after floating the samples as described in the experimental section. As can be seen, generally, the topographic patterns are preserved after the floating and redepositing of the film on a new substrate. This result indicates a symmetry of the pattern. The schematic of the procedure, including the selective swelling of the PVP block, formation of the topographic pattern after rapid evaporation of the solvent and local deformations of the PVP domains to adhere to the substrate during the deposition of the floated film is shown in Figure 5e.

**Figure 6:**
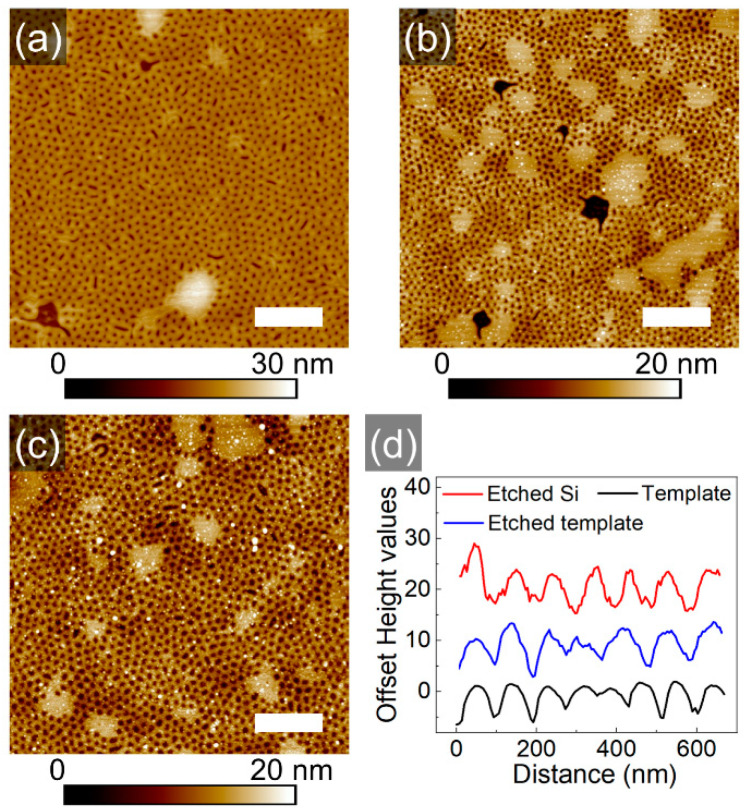
SFM topography images of the block copolymer template (**a**), of the etched sample (**b**) and of the transferred pattern (**c**). The scale bar is 1 μm. (**d**) Cross-sectional profiles of the samples, as indicated.

Further, the mesh-like block copolymer template as shown in Figure 4c, was etched into the underlying substrate via RIE, as described in the experimental section (Figure S8). Figure 6 displays SFM topography images of random areas of the initial template, of the sample after etching and after rinsing the etched sample in chloroform to remove residual polymer. The cross-sections of respective topography images illustrate the quality of the pattern transfer (Figure 6d). A quantitative analysis of the patterns by PSD Analysis and by averaging cross-sectional profiles of the topography scans is presented in Table 2. As can be seen from the data, the initial depth of the holes was successfully transformed. This is due to the high differences in the etching rates of the blocks (13 nm/min for PS and 29 nm/min for PVP) as well as due to the presumed segregation of the PVP to the bottom of the holes (schematic iv in Figure 5e). The critical dimensions (CD) of the transferred features are larger than those of the template. This result may indicate a gradient distribution of the PVP block within the walls of the meshes.

Further insights on the compatibility of the topographic and chemical patterns have been provided by selective metallization of the patterns. Immersion of the film into aqueous sodium tetrachloroplatinate solution leads to selective loading of PVP domains with [PtCl_4_]^2^ ions, which are converted into metalized structures upon treatment with oxygen plasma.

**Figure 7:**
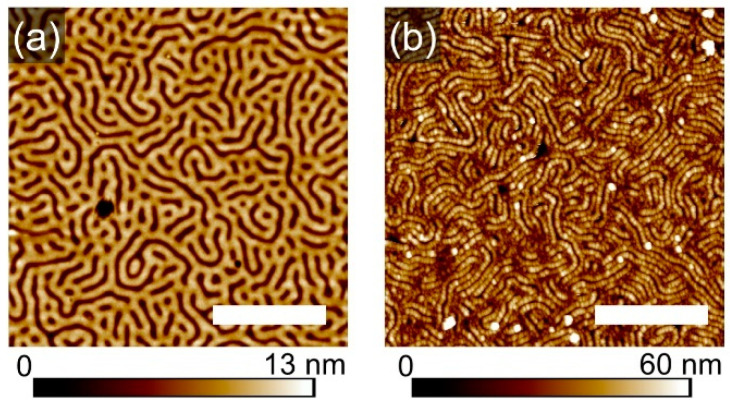

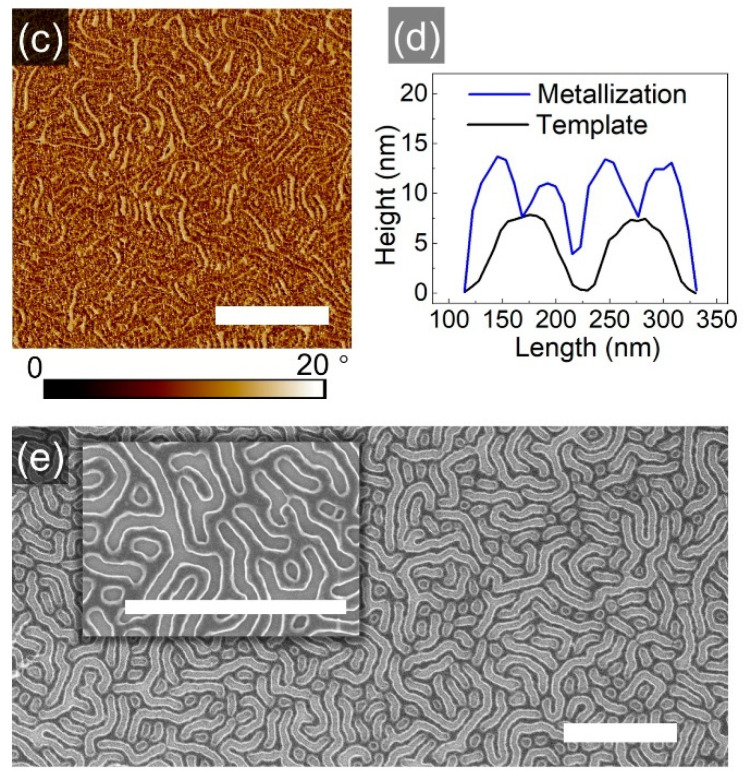
SFM topography (**a**,**b**) and phase (**c**) images of a striped template (**a**) and of respective metalized pattern (**c**). (**d**) Comparison of cross-section profiles of the structures. (**e**) SEM image of the metalized pattern with a close-up as inset. The scale bar in each image represents 1 μm.

Shown in Figure 7a,b are SFM images of the striped template (with feature dimensions ~110 nm) and of the respective metalized pattern, exhibiting wires with ~50 widths. As visualized in the cross-section profile in Figure 7d, the striped pattern is multiplicated, revealing that the PVP block was likely covering the walls of the stripes, while the PS block was segregated to the middle of the initial stripes. This kind of segregation and of the topographic pattern is supported by SEM images in Figure 7e, where the light-gray stripes indicate the higher metal fraction. The bright contour lines, which highlight the light-gray stripes, are typically attributed to artifacts, caused by the surface topography [40]. However, in this case, the SEM image can be considered as a confirmation of a non-homogeneous distribution of the PVP component within the geometrical pattern, demonstrating a mismatch between the topographic and chemical pattern. A plausible explanation of the development of such complex microphase separation was derived from resolving the morphological development on a shorter timescale.

### 3.4. Morphological Phase Transition on a Short Time Scale

**Figure 8:**
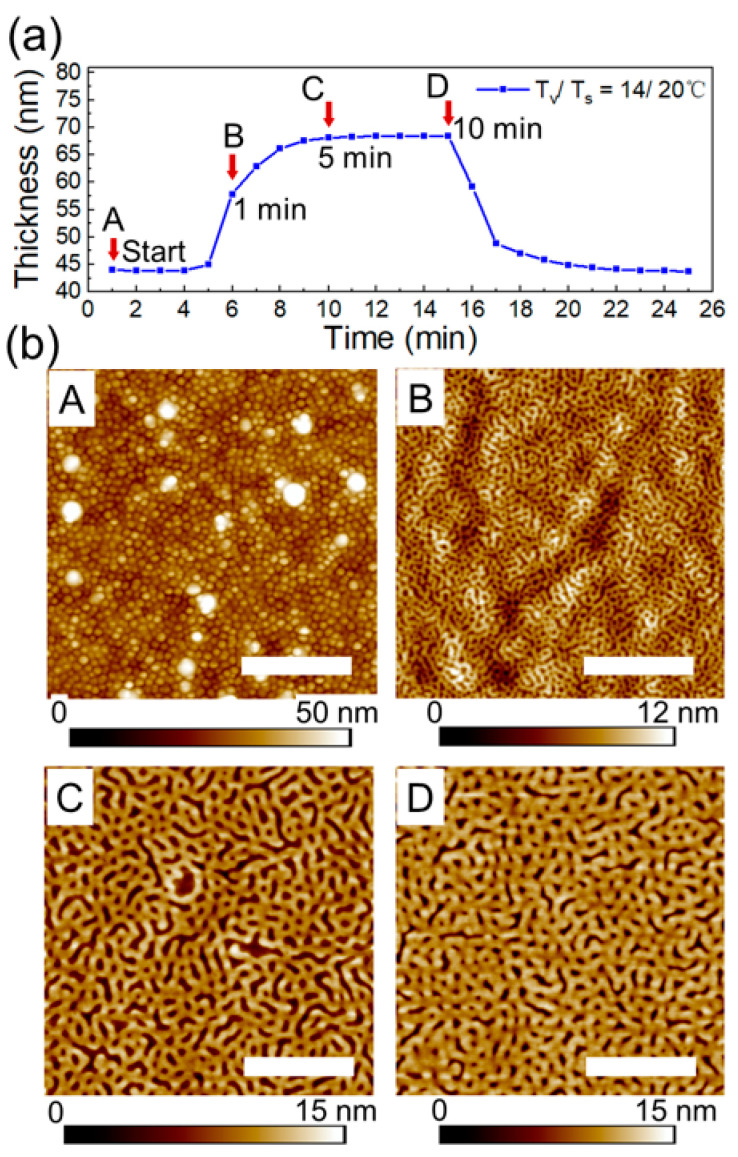
(**a**) Swelling kinetics of PS-PVP film annealed in 100% p/p_0_ chloroform vapors at T_v_/T_s_ = 14/20 °C. A, B, C and D correspond to the topography imagies of the PS-PVP film in (**b**). Arrows indicate the indicated annealing duration. (**b**) SFM topography images A, B, C, D of the surface structures of spin-coated film and of the samples after 1 min, 5 min and 10 min of exposure to the solvent vapors. The white scale bar in each image corresponds to 1 μm.

PS-PVP films used in this study were prepared by spin-coating from 1 wt% toluene solution. In toluene, which is a strongly selective solvent to PS block, PS-PVP chains form micelles with a poorly swollen PVP core and a highly swollen PS shell. The morphology of as-spin-coated films is represented by spherical structures with lateral dimensions of 70 ± 5 nm in a quenched state (Figure 8bA). Time-resolved swelling experiments have been conducted in the following way. Several samples have been prepared under identical conditions. The setup has been pre-equilibrated with the selected set of parameters T_v_/T_s_ = 14/20 °C, as described in the experimental section. The swelling of a 43 nm thick PS-PVP film was monitored for 10 min before the flow of the solvent vapor was stopped and the sample (designed as D, in Figure 8) was quenched. Then, similar procedures were performed with the other two samples for 1 min (sample B) and for 5 min (sample C) before quenching. Surface structures of the spin-coated film (sample A) and of the processed samples are presented in Figure 8b.

Taking into account the film thickness of ~40 nm, the initial structures in the spin-coated films can be assigned to hemispheres with the PVP core next to the substrate. Few larger objects seen in Figure 8 bA correspond to micelles with a larger aggregation number, which evolves in the solution upon aging. These larger micelles can be redispersed by heating and stirring the solution.

Upon rapid exposure to solvent vapor, the structure evolves into a mesh-like pattern. The transition occurs within 1 min, even if the maximum swelling is not reached. The characteristic dimensions of the mesh-like structures are comparable to the size of the initial spherical structures (Figure 8bB and Figure S9b). Remarkably, after 5 min of exposure to the vapors, a phase transition to a mesh-like pattern with doubled dimensions (about 133 nm, Figure S9g–i). The driving force of these transitions is presumably the coarsening of the interface between PS and PVP blocks, which is facilitated by the enhanced chain dynamics upon selective swelling of the PVP block. The observed two types of morphological transitions (A–B and B–C), as well as their dynamics are reproducible at other temperature sets, as shown in Figures S9 and S10 (Supplemental Information). We believe that the multiplication of the chemical pattern selective by metallization of the structures replicates the morphological transition (B–C).

## 4. Conclusions

Achieving high precision, reproducibility as well as high throughput processing of high-molecular-weight block copolymer templates in thin film is a challenging task. In this study, solvothermal annealing, including variation in the temperature of the substrate T_s_ and of the vapor T_v_, was employed to assess the relative contributions of the enhanced chain dynamics due to increased temperature or due to selective swelling on the resulting microphase-separated structures. The rearrangement of the initial morphology, frozen by spin-coating, proceeds on a timescale below one minute upon swelling in the vapors of selective solvent, independent of the applied annealing temperature. The (quasi)equilibrium dimensions of the structures are achieved on a time scale of minutes when the steady-state solvent uptake is reached. The long-range order of the microphase-separated patterns cannot be improved even after hours of annealing in solvent vapors.

Tunable microphase separation of high-molecular-weight lamella-forming polystyrene-b-poly(2vinylpyridine) block copolymer leading to mesh-like or striped topographic templates, suitable for the pattern transfer is achieved by setting T_v_/T_s_ combinations. The one-step formation of topographic patterns is presumably due to enhanced chloroform selectivity to the PVP block under strong confinement. The quenched patterns demonstrate a mismatch of the topographic and chemical patterns. This is manifested by the multiplication of the striped pattern upon selective metalization of the template and is attributed to the disclosed rapid morphological transition from initial non-equilibrium spherical morphology to the vertically oriented lamella. Apart from quantification of the solvothermal processing, the reported results disclose specific futures of microphase separation of high-molecular-weight block copolymers under strong confinement.

## Figures and Tables

**Table 1:** Dimensions of the patterns shown in Figure 4.

D_s_ = h_sw_/h_dry_	1.66	1.73	2.47	2.45
T_v_/T_s_ °C	14/20	24/30	19/20	29/30
CCD, nm	125 ± 29	115 ± 22	120 ± 32	111 ± 42
Depth, nm	6.2 ± 1.5	5.0 ± 1.0	4.7 ± 0.6	6.8 ± 0.5

**Table 2:** Dimensions of the template and transferred pattern shown and evaluated in Figure 6.

	Mask from Figure 6a	Transferred Pattern from Figure 6c
CCD	116 ± 22 nm	116 ± 23 nm
CD	65 ± 5 nm	74 ± 6 nm
Depth of holes	7 ± 1 nm	7 ± 1 nm

## Data Availability

The original contributions presented in the study are included in the article/Supplementary Materials, further inquiries can be directed to the corresponding author.

## Supplementary Materials

The following supporting information can be downloaded at: https://www.mdpi.com/article/10.3390/polym16131943/s1, Figure S1: Kinetic curves of stepwise swelling and deswelling of homopolymer and block copolymer films; Figure S2: Time-resolved swelling of PS-PVP films upon stepwise variation in T_s_ and T_v_. Figure S3: SAXS measurement of µm-thick PS-PVP film. Figures S4–S6: thickness-dependent morphological behavior of processed films. Figure S7: Power spectrum density analysis (PSDA) of the surface structures. Figure S8: Etching rates of the homopolymers. Figures S9 and S10: Swelling kinetics and time-resolved phase transitions.
