# Supplementary material for: Disclosing Topographical and Chemical Patterns in Confined Films of High-Molecular-Weight Block Copolymers under Controlled Solvothermal Annealing
Source: Polymers (Basel). 2024 Jul 8;16(13):1943. doi: 10.3390/polym16131943 (PMC11243801; doi:10.3390/polym16131943)
Supplement: Supplementary file 1 [file polymers-16-01943-s001.zip › polymers-3075307-supplementary.pdf]

SUPPLEMENTAL INFORMATION

Disclosing Topographical and Chemical Patterns in Confined Films of High-Molecular-Weight Block Copolymers under Controlled Solvothermal Annealing

Xiao Cheng [1,2], Jenny Tempeler [3], Serhiy Danylyuk [3], Alexander Böker [1] and Larisa Tsarkova [4,*]

[1] Fraunhofer Institute for Applied Polymer Research (IAP), Geiselbergstr. 69, 14476 Potsdam-Golm, Germany
[2] School of Civil Engineering, Southeast University, Dongnandaxue Road 2, Jiangning District, 211189 Nanjing, China
[3] Fraunhofer Institute for Laser Technology (ILT), Steinbachstr. 15, 52074 Aachen, Germany
[4] German Textile Research Center North-West (DTNW), Adlerstr. 1, 47798 Krefeld, Germany
* Correspondence: tsarkova@dtnw.de

Swelling under controlled vapor-flow conditions

Selectivity of the solvent

Figure S1 presents a time-resolved swelling and deswelling behavior of a PS-PVP block copolymer and respective homopolymer films when the partial vapor pressure p/p_0 in the chamber was stepwise adjusted by setting a corresponding mixture of a 100% p/p_0 vapor flow with a flow of dry nitrogen, by maintaining the temperature of the sample T_s at a constant level of 20°C and the temperature of the vapor T_s at 14°C. The degree of swelling at a steady state (typically, achieved after 20 min) has been used to evaluate the polymer volume fraction φ_p (a ratio of the dry film thickness to the swollen film thickness) as a function of p/p_0 (Figure S1 b). The polymer volume fraction (a reverse of the degree of swelling) in the PS film is systematically ~10% higher than that of the PVP homopolymer, clearly suggesting a selectivity of the chloroform towards a more polar polymer. Accordingly, the swelling of the PS-PVP block copolymer is defined by the contribution from both blocks, so the swelling curve of PS-PVP lies between the swelling curves of the homopolymer. Such systematic studies allow accessing the polymer-solvent interactions (χ, Flory-Huggins parameter) and comparing the selectivity of a given solvent to the block copolymer components. [1]

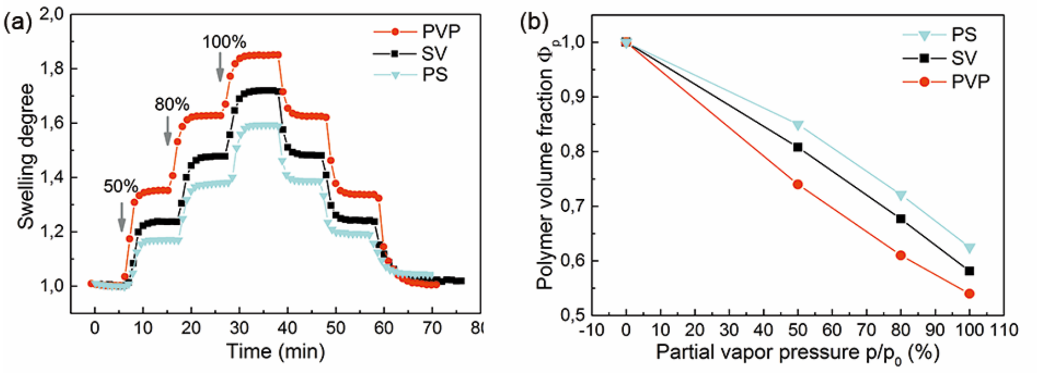

Figure S1. (a) Kinetic curves of stepwise swelling and deswelling of thin films of PS (green triangles), PVP (red circles) and PS-PVP (black squares). Dry film thickness was 48 nm for PS, 43 nm for PVP and 39 nm for PS-PVP films. Partial vapor pressure of chloroform p/p_0 was set to 50%, 80% and 100% as indicated by arrows. The swelling has been done at T_v = 14°C and T_s = 20°C. (b) Polymer volume fraction, ϕ at equilibrium swelling derived from the data in (a).

Swelling under variation of the temperatures of the vapors (T_v) and of the polymer film (substrate, T_s)

To explore the possibilities of our annealing setup to run solvothermal annealing in a reproducible and controlled manner, we analyzed the response of the solvent up-take by PS-PVP films to the changes in the temperatures of the vapors (T_v) and of the polymer film (substrate, T_s). Figure S2 a,b displays time-resolved swelling of PS-PVP films, when the partial vapor pressure of chloroform p/p_0 was kept constant at 100% and 50%, respectively. The changes in the solvent up-take were caused solely by the independent variation of the temperatures, T_s and T_v (right axis in plots in Figure S2 a,b), which were regulated by setting the targeted temperatures in respective thermostats. Simultaneously the changes of the film thickness are monitored with ellipsometry measurements (left axis in plots in Figure S2 a,b). By doing this, we wanted, on one hand to access the rate of the response of the solvent concentration in the chamber to the settings of the thermostat (the plotted temperature data were read from the data panel of the respective thermostat). On the other hand, such measurements comprise the dynamic response of the block copolymer film (dynamics of the solvent uptake) to the changes in the solvent vapor concentration. This response depends on the

solvent properties as well as on the block copolymer microstructure and chemical composition.

At the beginning of the measurements the conditions were preset as T_v = 20°C and T_s = 30°C for 15 min to equilibrate the vial with the solvent, the annealing chamber and the sample. At point A (A') the system was filled in with chloroform vapor. The swelling process was followed until steady state swelling was achieved, if possible. Then the next stepwise change of T_v or of T_s was undertaken.

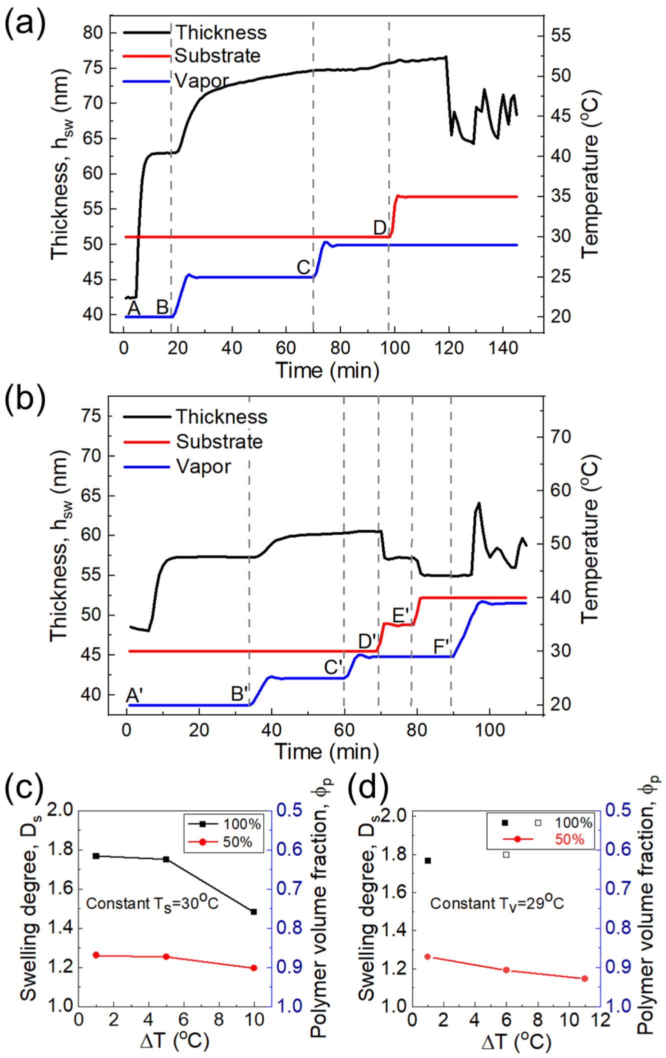

Figure S2. Time-resolved swollen thickness h_{sw} (left axis, black curves) of PS-PVP film upon stepwise increasing of T_s and T_v (right axis, red and blue curves, respectively) in (a) 100% and (b) 50% p/p_0 chloroform vapor. Temperature sets T_v/T_s are indicated by letters and correspond to the following conditions: (A,A') - 20°C/30°C; (B,B') - 25°C/30°C; (C,C') – 29°C/30°C; (D,D') – 29°C/35°C; (E') – 29°C/40°C; (F') 39°C/40°C. (c,d) Swelling degree D_s (left axis) and ϕ_p (right axis) as a function of the temperature difference ($\Delta T = T_s - T_v$) at constant T_v (c) and T_s (d) under 100% (black point) and 50% (red points).

As shown in Figure S2 a,b, the swelling process at 100% and 50% p/p_0 proceeds in a similar way up to conditions of 29°C/35°C (D,D'), with the degree of swelling, D_s reaching ~1.8 and ~1.2, respectively (Figure S2 c,d). A two-step mode is seen under conditions A(A') and B(B'), when ~90% of the maximum degree of swelling is achieved within 10- 20 min followed by a slow mode to achieve steady state. At point C(C'), the temperature of the vapor was further increased up to 29°C, still being 1°C lower than that of the substrate. However, no clear response of the polymer film could be measured. Possible reasons of such behavior could be attributed to solvent condensation inside tubing, as discussed earlier in [1].

At point D(D'), the temperature of the substrate T_s was increased to 35°C while keeping T_v at 29°C. The swelling behavior showed clear differences depending on p/p_0. In the case of 100% p/p_0 a slow increase in the film thickness was followed by instability in the system as a result of presumably condensation of the solvent on the polymer film, so that ellipsometry measurements became unreliable (Figure S2 a). In the case of 50% p/p_0 (Figure S2 b), increase in T_s was expectedly accompanied by the drop in the swollen thickness from 61 nm to 58 nm at steady state (drop in D' from 1.27 to 1.11). At point E', T_s was increased to 40 °C and the film responded by further decreases of swelling from 58 nm to 53 nm, however the system was stable and the steady state was achieved in less than 5 min. When T_v was increased to 39 °C (point F'), the experiment was no more reliable, most likely because of the condensation of the solvent.

Figure S2 c,d summarizes the results in terms of swelling degree, D_s (polymer volume fraction ϕ_p) and $\Delta T=T_s-T_v$ with T_v constant at 29°C and T_s constant at 30 °C, respectively. These results allowed us to select solvothermal conditions for the annealing experiments, in order to evaluate the combined plasticizing effect of solvent molecules and of the elevated temperature on the chain dynamics and on the evolutions of the microphase separation of PS-PVP under strong confinement. In particular, further experiments have been performed at varied T_v/T_s and at 100% p/p_0, which provided a stability of the annealing system and, as well as, a high solvent concentration in the vapor.

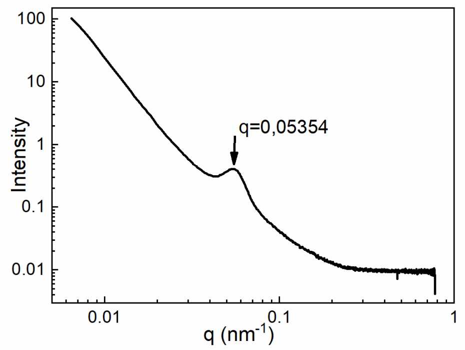

Figure S3. SAXS measurement of μ-thick PS-PVP film. Peak at q=0.05354 indicates a domain spacing L_0 of 117.3 nm.

Thickness-dependent morphological behavior

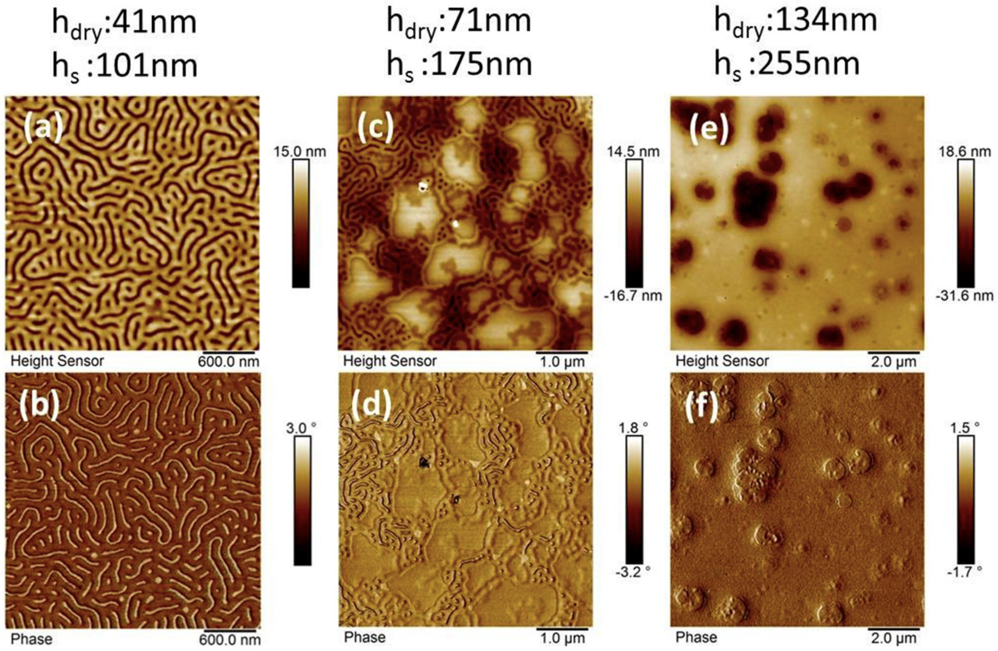

Figure S4. SFM (a) (c) (e) height and (b) (d) (f) phase images of PS-PVP films annealed in chloroform vapors at T_v /T_s =19 /20 °C for 200 min with a degree of swelling of 2.5.

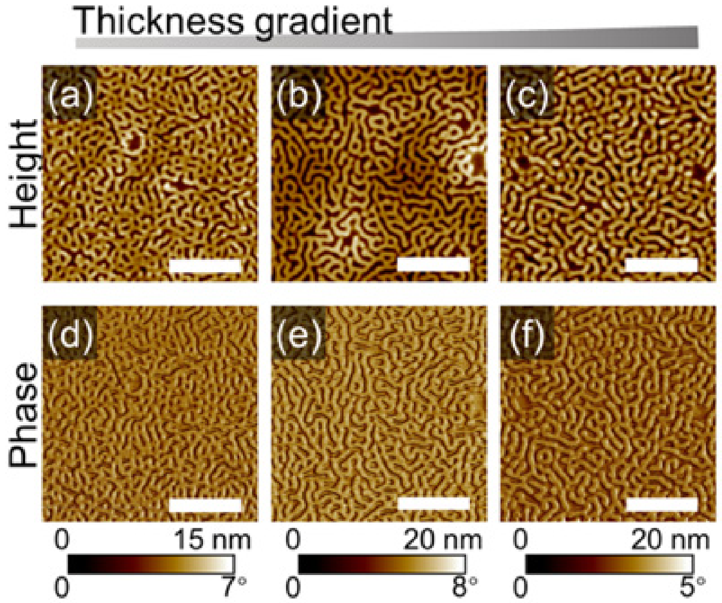

Figure S5. SFM topography (a, b, c) and corresponding phase (d,e,f) images of 40 nm-thick PS-PVP film annealed under 100% chloroform vapor at T_v /T_s =14 /20°C swelling degree of ~1.7 for 5 min. The images from left to right were scanned from the middle towards the edge of the sample, resulting in a slight thickness gradient. The scale bar in each image corresponds to 1 µm.

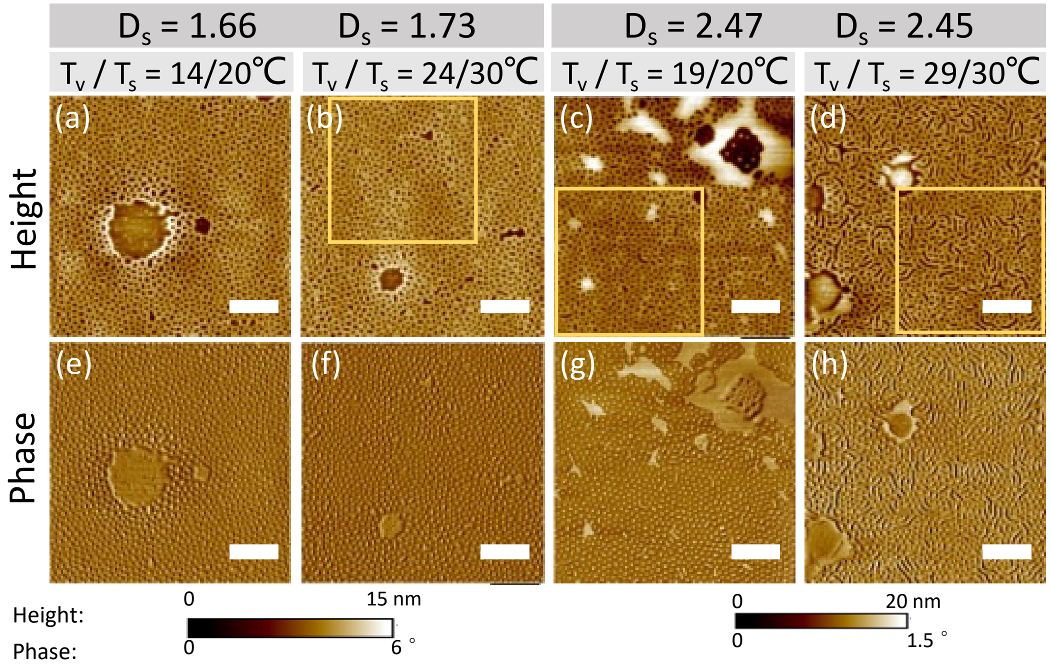

Figure S6. SFM topography and phase images of PS-PVP films with h_{dry}= 42±2 nm annealed under indicated T_v/T_s and 100% p/p_0 of chloroform vapor for 200 min. Yellow squares mark regions of respective zoom-in below the image. Yellow squares mark regions of respective zoom-in images in Figure 3 of the paper. The white scale bar in each image corresponds to 1 μm. Images (a, c and d) are reprinted from Ref. 1 with permission.

The holes of lower terraces with a structureless wetting layer in Figure S6 a, b indicate that the film thickness is slightly lower than a monolayer of the PL phase. In contrast, featureless regions with a thickness that is higher than PL structures in Figure S6c indicate a local formation of in-plane lamella phase when the swollen thickness approaches L_0.

Characterization of the patterns

Power Spectral Density (PSD) plots of the SFM images in Figure 4a'-d' are presented in Figure S5. The XZ data were exported from the Power Spectral Density histogram, which was computed through "2D Isotropic" mode under PSD function in Nanoscope Analysis 3.0 software. As the spatial frequency is the inverse of the wavelength of the roughness features, the domain spacing of periodic features can be calculated according to the frequency corresponding to 1st peak power spectral density. To get the deviations in the dimensions, the PSD curves were fitted to Gaussian function and then subtracted

(or added) the half width at half maximum from (to) frequency at peaks. The corresponding center-to-center distances (CCDs) and the deviations are shown in Table 1.

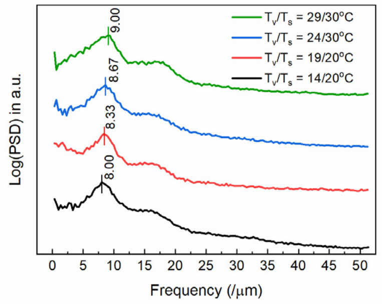

Figure S7. Power spectrum density(PSD) analysis using Nanoscope Software of the images of the surface structures of the processed films as in Figure 4.

Etching behavior

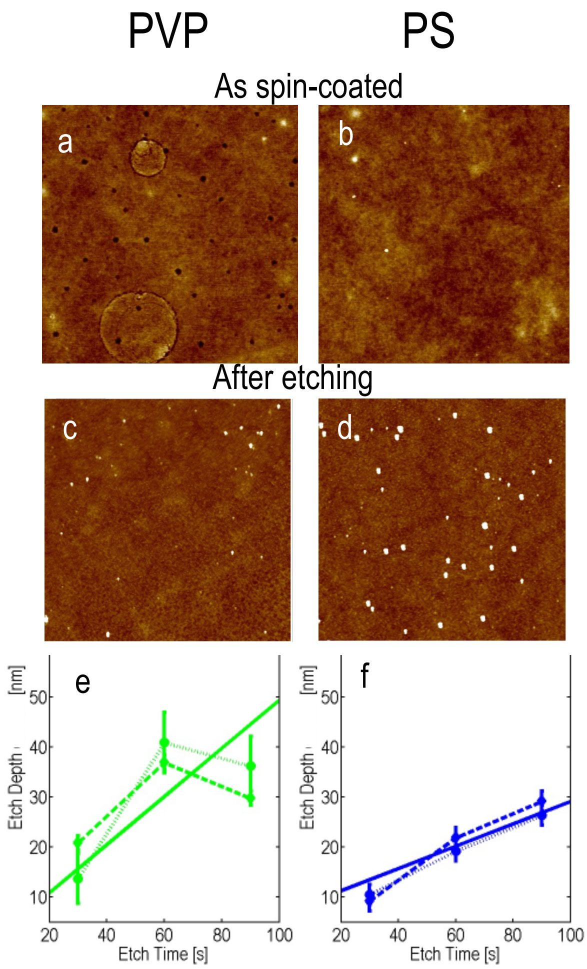

Figure S8. (a-d) SPM topography images (3 x 3 μm2) of thin films pf PVP and PS homopolymers before and after etching. (e,f) Etching rate of PVP and PS, resulting in averaged values of 29 nm/min and 13 nm/min (solid lines), respectively.

Time-evolution of the morphological behavior

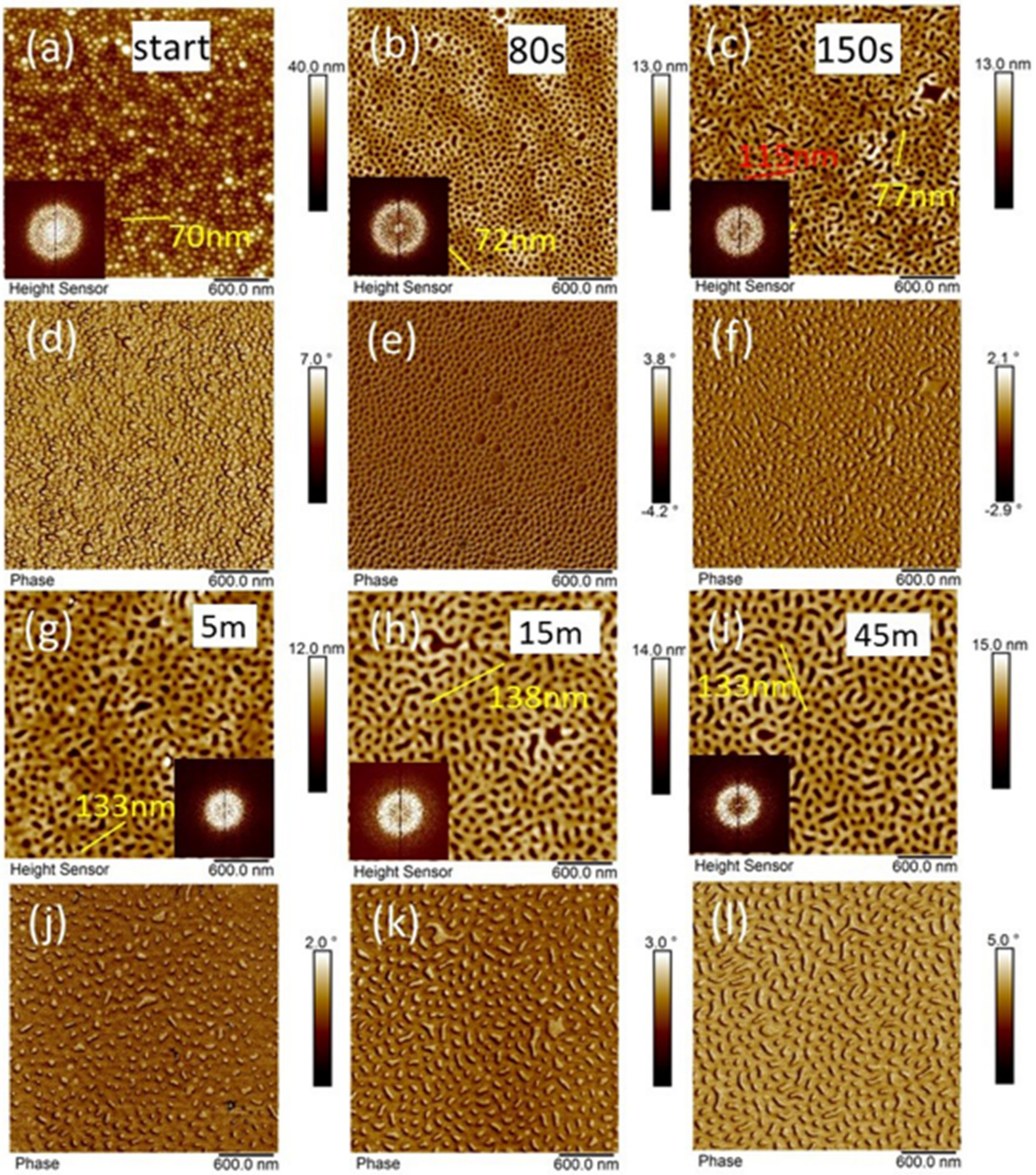

Figure S9. SFM height images (a-c, g-i) and their corresponding phase images (d-f, j-l) representing time-resolved morphology of PS-PVP film with h_{dr} ~40 nm annealed in 100% p/p_0 chloroform vapors at T_v /T_s =19/20°C. Insets are Fast Fourier Transform (FFT) transfer images, which indicate the quality of a long-range order. The domain periods in each topography image have been evaluated from the topographic cross-sections along yellow lines.

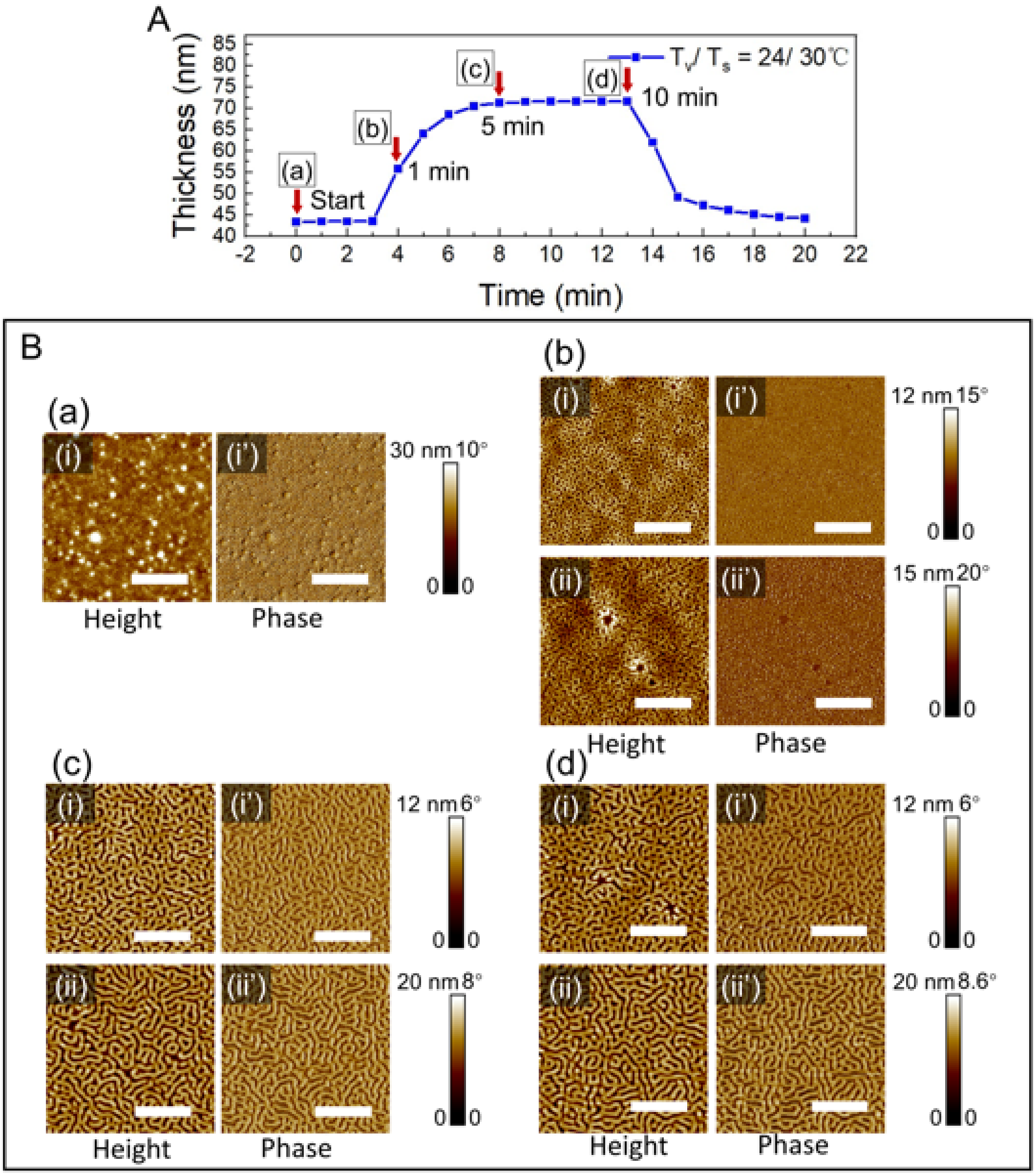

Figure S10. A: Swelling kinetics of PS-PVP film annealed in 100% *p/p₀* chloroform vapors at T_V /T_S =24 /30°C. B: SFM images of the surface structures of as spin-coated film (a) and of the samples after 1 min (b), 5 min (c) and 10 min (d) of exposure to the solvent vapors. The white scale bar in each image corresponds to 1 µm.

References:

1. Cheng, X.; Boeker, A.; Tsarkova, L., Temperature-controlled solvent vapor annealing of thin block copolymer films. *Polymers* **2019,** *11*, 1312pp.
